# Direct health-care costs attributed to hip fractures among seniors: a matched cohort study

**DOI:** 10.1007/s00198-012-2034-6

**Published:** 2012-06-27

**Authors:** M. Nikitovic, W. P. Wodchis, M. D. Krahn, S. M. Cadarette

**Affiliations:** 1 Leslie Dan Faculty of Pharmacy, University of Toronto, 144 College Street, Toronto, ON M5S 3M2 Canada; 2 Institute for Clinical Evaluative Sciences, Toronto, ON Canada; 3 Toronto Health Economics and Technology Assessment (THETA) Collaborative, University of Toronto, Toronto, ON Canada; 4 Department of Health Policy, Management and Evaluation, University of Toronto, Toronto, ON Canada; 5 Toronto Rehabilitation Institute, Toronto, ON Canada

**Keywords:** Canada, Direct service costs, Hip fractures, Osteoporosis

## Abstract

**Summary:**

Using a matched cohort design, we estimated the mean direct attributable cost in the first year after hip fracture in Ontario to be $36,929 among women and $39,479 among men. These estimates translate into an annual $282 million in direct attributable health-care costs in Ontario and $1.1 billion in Canada.

**Introduction:**

Osteoporosis is a major public health concern that results in substantial fracture-related morbidity and mortality. It is well established that hip fractures are the most devastating consequence of osteoporosis, yet the health-care costs attributed to hip fractures in Canada have not been thoroughly evaluated.

**Methods:**

We determined the 1- and 2-year direct attributable costs and cost drivers associated with hip fractures among seniors in comparison to a matched non-hip fracture cohort using health-care administrative data from Ontario (2004–2008). Entry into long-term care and deaths attributable to hip fracture were also determined.

**Results:**

We successfully matched 22,418 female (mean age = 83.3 years) and 7,611 male (mean age = 81.3 years) hip fracture patients. The mean attributable cost in the first year after fracture was $36,929 (95 % CI $36,380–37,466) among women and $39,479 (95 % CI $38,311–$40,677) among men. These estimates translate into an annual $282 million in direct attributable health-care costs in Ontario and $1.1 billion in Canada. Primary cost drivers were acute and post-acute institutional care. Approximately 24 % of women and 19 % of men living in the community at the time of fracture entered a long-term care facility, and 22 % of women and 33 % of men died within the first year following hip fracture. Attributable costs remained elevated into the second year ($9,017 among women, $10,347 among men) for patients who survived the first year.

**Conclusions:**

We identified significant health-care costs, entry into long-term care, and mortality attributed to hip fractures. Results may inform health economic analyses and policy decision-making in Canada.

## Introduction

Osteoporosis is a major public health concern that results in substantial fracture-related morbidity and mortality [1–3]. An estimated 30,000 hip fractures occur annually in Canada, with incidence projected to increase with our aging population [4]. It is well established that hip fractures are the most devastating consequence of osteoporosis, yet the health-care costs attributed to hip fractures in Canada have not been thoroughly evaluated. Prior Canadian cost-of-illness studies are outdated [5] or limited [6, 7]. Comprehensive Canadian health-care costs attributed to hip fractures are needed to inform health economic analyses and guide policy decisions related to health resource allocation [8]. The main objective of our study was to determine the mean sex-specific direct health-care costs and outcomes attributable to hip fractures in Ontario seniors over a 1- and 2-year period.

## Methods

We used a matched cohort study design that leveraged Ontario health-care administrative databases to determine the 1- and 2-year costs attributed to hip fractures. In Ontario, medical claims data are available for all residents, and pharmacy claims are available for seniors (age ≥65 years) under the Ontario Drug Benefit (ODB) program. We identified all hip fractures between April 1, 2004 and March 31, 2008 based on hospital claims. In-hospital diagnostic codes for hip fracture have been well validated, with estimated sensitivity and positive predictive values of 95 % [9–11]. The first date of hip fracture diagnosis defined the index date. To allow for a minimum 1 year pre-fracture drug exposure period, we excluded those aged less than 66 years at index. We restricted inclusion to incident fractures by excluding patients with any prior diagnosis of hip fracture since April 1991, the first date of available data. To maximize the likelihood that hip fractures were due to underlying low bone mineral density attributed to osteoporosis, we excluded those with a trauma code identified within 7 days of index and patients with: malignant neoplasm, Paget's disease diagnosis, or non-osteoporosis formulations of bisphosphonates or calcitonin within the year prior to index. Finally, we excluded non-Ontario residents and those with death identified prior to index.

We employed an incidence density sampling strategy to identify non-hip fracture matches. First, a random index date was assigned to all persons in Ontario according to the sex-specific distribution of index dates among the hip fracture cohort. Second, the same exclusion criteria applied to the hip fracture cohort were applied. Individuals were also excluded from the non-hip fracture cohort if they had a hip fracture on or within 2 years after their assigned index date. Third, all eligible individuals in the hip fracture cohort were matched on index date (month and fiscal year), age (±3 months), sex, and residence status (community vs. long-term care (LTC)) to non-hip fracture patients. Fourth, a propensity score for hip fracture was calculated using logistic regression according to collapsed aggregated diagnostic group (comorbidity score) [12], rurality index for Ontario (population density and access to health-care services score) [13], and income quintile. Finally, hip fracture patients were matched 1:1 to non-hip fracture individuals on the logit of the propensity score using a greedy matching algorithm with a maximum caliper width of 0.2 and no replacement [14]. We therefore hard matched on age, sex, and residence status at index; all factors for which we were interested in providing stratified results; and then propensity score matched on comorbidity and sociodemographics that may impact health-care resource utilization.

### Health-care costing and outcomes

We used an Ontario health-care payer perspective, where only direct costs paid by the Ontario Ministry of Health and Long-Term Care were considered. When possible, all costs were applied based on the year they were incurred and then inflated and reported in 2010 Canadian dollars using the health-care component of the Ontario consumer price index (CPI, www.statscan.gc.ca). Detailed methods for case-costing using administrative databases in Ontario have recently been published [15]. In brief, *acute hospitalizations*, *emergency department*, and s*ame day surgery* costs were calculated using the resource intensity weight method that uses the average provincial costs per weighted case based on distinct case mix groups [16, 17]. Costing in *complex continuing care* was based on distinct resource utilization groups, case mix index, and number of days in care [18]. *Physician service* costs and *prescription drug* costs were based on the total amount paid to the physician/pharmacy from the Ministry of Health. Costs related to length of stay in *rehabilitation* were based on the rehabilitation patient group case mix classification and weighting system for Ontario [19–21]. Costs for *home care* were determined by applying an average cost per service (or hour) [22]. *LTC* costs were calculated based on the average cost per day and length of stay. In addition to health-care costs, we assessed the number of individuals who died, entered LTC, and experienced a second hip fracture.

### Statistical analysis

Cohort characteristics were summarized using means and proportions. Balance between matched cohorts was assessed using standardized difference, where values <0.1 indicate balance [23]. Cohorts were compared for each matched variable as well as on osteoporosis screening (DXA test), diagnosis, treatment, and fractures (humerus/radius/ulna, vertebral, or others) within 365 days prior to index.

Health resource utilization and outcomes were compared between matched cohorts using the McNemar chi-square test for categorical variables and the paired *t* test for continuous variables. Total costs were determined by summation of each costing component and presented as the mean cost over the first and second year. Attributable hip fracture costs were determined by subtracting costs in the non-hip fracture cohort from the costs in the matched hip fracture cohort [24]. Variance estimation (95 % CI) was determined using bootstrapping with replacement [24]. All costs were stratified by resource type (acute hospitalization, same day surgery, emergency department, complex continuing care, rehabilitation, LTC, home care, physician services, prescriptions for osteoporosis, and pain medications), sex, age group (66–69, 70–74, 75–79, 80–84, 85–89, 90+), and residence status (community or LTC) at baseline. In an effort to determine costs attributed to death from hip fracture, we further evaluated costs among concordant pairs who survived or died within 1- and 2-years of follow-up. One-year attributable hip fracture costs in Canada were estimated by multiplying sex-specific attributable mean costs in Ontario by 30,000—the total number of hip fractures estimated to occur annually in Canada [4, 25].

## Results

We identified 36,253 hip fracture patients, of which 31,064 (86 %) were eligible. Exclusions were primarily as a result of prior hip fracture (56 % females and 30 % males) and a diagnosis of malignant neoplasm (34 % females, 52 % males), Appendix Fig. 1. After applying exclusion criteria and identifying suitable non-hip fracture matches, the final cohort included 30,029 matched pairs (22,418 females, 7,611 males). Mean age at hip fracture was 83.3 years (SD = 7.1) for females and 81.3 years (SD = 7.1) for males (Table 1). About one-fifth (21 % females, 18 % males) of patients resided in LTC at the time of fracture. The sex-specific matched fracture and non-hip fracture cohorts were well balanced on matched variables, as well as on prior osteoporosis diagnosis. However, more hip fracture patients had been dispensed an osteoporosis medication or incurred a non-hip fracture in the year prior to fracture.

**Fig. 1:**
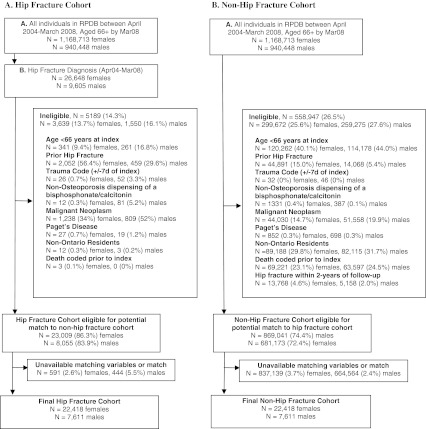
Study flow diagram for hip and non-hip fracture cohort inclusion. *RPDB* means registered persons database. Exclusions are not mutually exclusive and thus will not add to 100 %

**Table 1:** Baseline characteristics of hip fracture cohort and matched non-hip fracture cohort

Variable	Value	Females					Males				
		Hip fracture (*N* = 22,418)		Non-hip fracture (*N* = 22,418)		SD	Hip fracture (*N* =7,611)		Non-hip fracture (*N* = 7,611)		SD
		*N*	%	*N*	%		*N*	%	*N*	%	
Age	Mean ± STD	83.3 ± 7.1		83.3 ± 7.1		0	81.3 ± 7.1		81.3 ± 7.1		0
	66–69	869	3.9	869	3.9	0	483	6.3	483	6.3	0
	70–74	1,893	8.4	1,893	8.4	0	940	12.4	940	12.4	0
	75–79	3,564	15.9	3,564	15.9	0	1,624	21.3	1,624	21.3	0
	80–84	5,802	25.9	5,802	25.9	0	1,897	24.9	1,897	24.9	0
	85–89	5,775	25.8	5,775	25.8	0	1,685	22.1	1,685	22.1	0
	90+	4,515	20.1	4,515	20.1	0	982	12.9	982	12.9	0
Fiscal year	04/05	5,786	25.8	5,786	25.8	0	1,856	24.4	1,856	24.4	0
	05/06	5,481	24.4	5,481	24.4	0	1,871	24.6	1,871	24.6	0
	06/07	5,539	24.7	5,539	24.7	0	1,919	25.2	1,919	25.2	0
	07/08	5,612	25.0	5,612	25.0	0	1,965	25.8	1,965	25.8	0
RIO^a^	Mean ± STD, 0 (most urban) to 100 (most rural)	16.7 ± 18.9		16.1 ± 18.7		0.03	17.3 ± 19.6		17.1 ± 20.1		0.01
LTC^a^		4,797	21.4	4,797	21.4	0	1,352	17.8	1,352	17.8	0
Income quintile^a^	1 (low)	5,218	23.3	5,315	23.7	0.01	1,739	22.8	1,649	21.7	0.03
	2	4,536	20.2	4,563	20.4	0	1,569	20.6	1,625	21.4	0.02
	3	4,361	19.5	4,377	19.5	0	1,419	18.6	1,417	19.3	0.02
	4	4,216	18.8	4,119	18.4	0.01	1,421	18.7	1,396	18.3	0.01
	5 (high)	4,087	18.2	4,044	18.0	0	1,463	18.0	1,470	19.3	0
Number of CADGs^b^	0–3	8,079	36	8,032	35.8	0	2,502	32.9	2,360	31	0.04
	4–7	13,567	60.5	13,670	61	0.01	4,816	63.3	4,987	65.5	0.05
	8–12	772	3.4	716	3.2	0.01	293	3.8	264	3.5	0.02
Osteoporosis diagnosis^b^		2,050	9.1	1,785	8.0	0.04	271	3.6	180	2.4	0.07
DXA test^b^		2,346	10.5	2,707	12.1	0.05	337	4.4	296	3.9	0.03
Osteoporosis treatment^b^		7,145	31.9	6,178	27.6	0.1^c^	753	9.9	448	5.9	0.15^c^
Prior fracture^b^											
Humerus/radius/ulna		948	4.2	464	2.1	0.12^c^	183	2.4	58	0.8	0.13^c^
Vertebral		329	1.5	110	0.5	0.1^c^	87	1.1	36	0.5	0.07
Other^d^		2,863	12.8	493	2.2	0.41^c^	903	11.9	134	1.8	0.41^c^

*CADG* collapsed ambulatory diagnostic group, *DXA* dual-energy X-ray absorptiometry, *IQR* interquartile range, *LTC* long-term care, *RIO* rurality index for Ontario, *SD* standardized difference, *STD* standard deviation

^a^Based on postal code and census data at time of index

^b^Medical and pharmacy claims identified within 365 days prior to index

^c^SD >0.1 indicates unbalance between cohorts [23]

^d^Other = femur, pelvis, lumbar spine, ribs, shoulder and upper arm, shoulder girdle, pathological or stress fracture

### Outcomes and resource utilization 

With the exception of same day surgery, more individuals in the fracture cohort than the non-hip fracture cohort utilized health-care resources (Table 2). Approximately 30 % of hip fracture patients required rehabilitation, 18 % required complex continuing care, and over 65 % received home care services. Physician services were utilized by more than 94 % of individuals in both cohorts; however, hip fracture patients received nearly twice as many individual services: 2,468,744 vs. 1,336,071 (women) and 852,834 vs. 480,829 (men). Of patients residing in the community at the time of hip fracture, 19 % (men) to 24 % (women) entered a LTC facility within a year of hip fracture. Less than 22 % of men and 50 % of women received osteoporosis medication after hip fracture. More men (33 %) than women (22 %) died within the year after hip fracture, resulting in an absolute increase in death of 23 % among men and 13 % among women in the first year. Risk of death increased with age and LTC residence at baseline.

**Table 2:** Health resource utilization and outcomes in first year after hip fracture compared to matched non-hip fracture cohort, by sex

	Females			Males		
	Percent hip fracture cohort (*N* = 22,418)	Percent non-hip fracture cohort (*N* = 22,418)	Percent attributable	Percent hip fracture cohort (*N* = 7,611)	Percent non-hip fracture cohort (*N* = 7,611)	Percent attributable
Resource utilization						
Acute hospitalizations	100	19.1	80.9^*^	100	22.6	77.4^*^
Same day surgeries	9.5	12.8	−3.3^*^	14.1	19.4	−5.3^*^
Emergency visits	86.5	36.6	49.9^*^	86.3	38.1	48.2^*^
Complex continuing care	17.7	1.4	16.3^*^	17.8	1.4	16.4^*^
Rehabilitation	32.8	1.4	31.4^*^	31.9	1.1	30.8^*^
Long-term care	38.0	24.6	13.4^*^	30.0	20.1	9.9^*^
Community at index	23.6	4.6	19.0^*^	19.0	3.4	15.6^*^
Home care	69.5	26.3	43.2^*^	66.1	21.5	44.6^*^
Physician services	100	94.5	5.5^*^	100	94.7	5.3^*^
DXA test	4.3	5.2	−0.9^*^	2.5	1.9	0.6^*^
Prescriptions	92.4	93.2	−0.8^*^	85.3	92.1	−6.8^*^
Osteoporosis treatment	43.7	27.8	15.9^*^	21.7	6.6	15.1^*^
Opioids	53.7	28.2	25.5^*^	48.7	24.9	23.8^*^
NSAIDs	18.9	23.4	−4.5*	17.2	22.6	−5.4^*^
Health outcomes						
Second hip fracture	1.2	0	1.2^*^	0.8	0	0.8^*^
Death (overall)	22.2	9.3	12.9^*^	33.4	10.8	22.6^*^
Age group						
66–69	9.3	1.7	7.6^*^	13.2	1.9	11.3^*^
70–74	11.7	2.4	9.3^*^	19.2	3.9	15.3^*^
75–79	14.1	4.4	9.7^*^	26.9	6.8	20.1^*^
80–84	18.9	7.3	11.6^*^	33.2	10.1	23.1^*^
85–89	25.1	11.1	14.0^*^	43.3	16.3	27.0^*^
90+	35.9	17.7	18.2^*^	51.6	20.5	31.1^*^
LTC at index	37.0	22.6	14.4^*^	53.6	28.9	24.7^*^
Community at index	18.2	5.7	12.5^*^	29.1	6.9	22.2^*^

*Attributable* percentage of hip fracture patients − percentage of non-hip fracture patients, *LTC* long-term care, NSAID nonsteroidal anti-inflammatory drug

* *p* < 0.05 (significant at this level)

During the 2-year follow-up period, 3 % of females and 2 % of males incurred a subsequent hip fracture. Health services utilization in the second year remained elevated in the hip fracture cohort. Among those who survived the first year, a marginal increase in death of 3 % for women and 6 % for men in the hip fracture cohort was observed in the second year (Appendix Table 5).

### Health-related costs

The total direct 1-year health-care cost of hip fracture ranged from $52,232 (females) to $54,289 (males) with mean 1-year attributable cost of $36,929 for females and $39,479 for males (Table 3). Applying these sex-specific mean costs to the estimated 30,000 hip fractures that occur annually in Canada (75 % among women), the direct attributable health-care cost of hip fracture is approximately $1.1 billion per year in Canada. Acute hospitalizations accounted for the largest component of attributable hip fracture costs, with 38 %–41 % of the cost resulting from the index hospitalization. Other primary drivers of first year costs included complex continuing care, rehabilitation, and physician services. Attributable costs generally decreased with age, reflecting both increased total costs with age in the non-hip fracture cohort and increased risk of death after hip fracture. Costs among the hip fracture cohort remained elevated into the second year post fracture, with a mean attributable cost of $4,599 for females and $3,083 for males (Appendix Table 6).

**Table 3:** Mean total direct health-care costs (2010 Canadian dollars) in first year after index date in the hip fracture and non-hip fracture cohorts, by sex

Resource type	Females (*N* = 22,418)				Males (*N* = 7,611)			
	Hip fracture	Non-hip fracture	Attributable (95 % CI)	%	Hip fracture	Non-hip fracture	Attributable (95 % CI)	%
Acute hospitalizations	21,502	2,710	18,792 (18,471, 19,119)	51	24,915	3,626	21,289 (20,573, 21,957)	54
Index hospitalization	14,210	–	14,210 (14,021, 14,400)	39	16,158	–	16,158 (15,711, 16,605)	41
Same day surgeries	120	153	−33 (−44, −22)	0	178	236	−58 (−83, −37)	0
Emergency visits	769	286	483 (472, 495)	1	831	322	509 (486, 532)	1
Complex continuing care	5,996	408	5,588 (5,323, 6,872)	15	6,934	466	6,468 (5,859, 7,037)	16
Rehabilitation	5,518	268	5,250 (5,107, 5,396)	14	5,700	247	5,453 (5,184, 5,730)	14
Long-term care	9,419	6,949	2,470 (2,315, 2,654)	7	6,746	5,494	1,252 (956, 1,521)	3
Home care	2,132	997	1,135 (1,069, 1,149)	3	2,050	705	1,345 (1,235, 1,458)	4
Physician services	4,525	1,422	3,103 (3,065, 3,142)	9	4,905	1,640	3,265 (3,190, 3,353)	8
Prescription medications	2,251	2,111	140 (102, 177)	0	2,030	2,073	−43 (−113, 34)	0
Total mean cost/year	52,232	15,303	36,929 (36,380, 37,466)	100	54,289	14,810	39,479 (38,331, 40,677)	100
Age group								
66–69	45,886	7,020	38,866 (35,910, 41,608)		46,551	6,699	39,852 (35,439, 44,764)	
70–74	47,250	9,373	37,877 (36,063, 39,850)		52,446	9,568	42,878 (39,501, 46,073)	
75–79	50,924	12,437	38,487 (37,222, 38,489)		56,927	14,549	42,378 (39,472, 45,240)	
80–84	52,863	14,859	38,004 (36,939, 39,111)		55,739	16,186	39,553 (37,312, 41,752)	
85–89	54,542	17508	37,034 (36,023, 38,131)		54,456	16,647	37,809 (35,510, 40,251)	
90+	52,810	19,396	33,414 (32,119, 34,693)		52,405	18,433	33,972 (31,164, 36,869)	

*Attributable* mean cost hip fracture cohort − mean cost non-hip fracture cohort, *CI* confidence interval

Mean total and attributable hip fracture costs stratified by residence status, number of hip fractures, and survival status are summarized in Table 4. Attributable costs were greatest among individuals residing in the community at baseline, those incurring a second hip fracture, and those who survived the study period. Among matched survivors, the mean 1-year attributable costs were $41,149 in females and $45,742 in males. First-year attributable costs among those who died in the first year were $10,935 among women and $14,451 among men. Among individuals who survived the first year, second-year attributable costs were $9,017 for women and $10,347 for men. LTC residence, acute hospitalizations, and home care accounted for the greatest proportion of the latter costs.

**Table 4:** Total and attributable direct health-care costs (2010 Canadian dollars) of hip fracture stratified by residence at baseline, second hip fracture, and survival

Cost stratification	Females				Males			
	%^a^	Hip fracture mean ($)	Non-hip fracture mean ($)	Attributable mean ($) (95 % CI)	%^a^	Hip fracture mean ($)	Non-hip fracture mean ($)	Attributable mean ($) (95 % CI)
Residence status (cost year 1)								
LTC at baseline	100	48,723	37,510	11,213 (10,469, 11,986)	100	46,931	37,758	9,174 (7,365, 10,911)
Community at baseline	100	53,187	9,258	43,930 (43,292, 44,560)	100	55,878	9,853	46,025 (44,821, 47,313)
LTC at baseline—survived year 1^b^	78.4	59,015	41,474	17,541 (16,803, 18,218)	73.7	63,159	42,895	20,263(18,163, 22,577)
Community baseline—survived year 1^b^	94.8	53,386	8,139	45,247 (44,548, 45,955)	93.9	56,750	8,184	48,566 (47,118, 50,174)
Second hip fracture								
Second hip fracture in year 1 (cost year 1)	NA	85,614	14,992	70,621 (65,777, 76,063)	NA	87,726	14,088	73,638 (60,853, 86,245)
Second hip fracture in year 2 (cost year 2)	NA	52,912	13,018	39,895 (36,459, 43,374)	NA	63,939	11,481	52,458 (44,611, 60,923)
Survival status^b^								
Survived year 1 (cost year 1)	96.3	54,218	13,069	41,149 (40,489, 41,774)	91.4	57,390	11,648	45,742 (44,257, 47,098)
Survived year 1 (cost year 2)	96.3	22,983	13,966	9,017 (8,578, 9,471)	91.4	22,909	12,563	10,347 (9,417, 11,275)
Survived year 2 (cost year 2)	80.1	22,019	12,467	9,552 (9,141, 10,004)	71.7	21,032	10,524	10,507 (9,514, 11,451)
Died year 1 (cost year 1)	13.8	34,873	23,938	10,935 (8,347, 13,364)	15.2	40,216	25,765	14,451 (10,062, 18,826)
Died year 2 (cost year 2)	10.5	23,696	23,470	226 (−4,297, 4,939)	11.2	26,806	26,336	469 (−5,073, 6,383)
Died year 2 (cost year 1)	10.5	70,601	32,134	38,466 (36,376, 39,487)	11.2	72,568	26,336	46,232 (38,285, 47,503)

*Attributable* mean cost hip fracture cohort − mean cost non-hip fracture cohort, *CI* confidence interval, *LTC* long-term care, *NA* not applicable

^a^Percentage of hip fracture patients with a matched concordant pair

^b^Calculated only among concordant pairs who both survived or died in the given year

Across the four fiscal years evaluated, the total cumulative first year attributable cost of hip fractures in Ontario was estimated at $282.1 million (females = $206.9 million, males = $75.1 million). The total cumulative attributable cost in the second year was $64.5 million in Ontario.

## Discussion

Our results emphasize the major health and economic burden of hip fractures on the Canadian health-care system. The 1 year direct attributable health-care system cost of hip fracture was $282.1 million in Ontario, with survivors costing an additional $64.5 million in the second year post-fracture. Based on these estimates and reports that indicate approximately 30,000 hip fractures annually in Canada [4, 25], the direct attributable health-care cost of hip fracture is approximately $1.1 billion per year in Canada.

Three prior studies have evaluated the longitudinal cost of hip fractures from a Canadian perspective [5–7]. The first study estimated the mean total 1-year direct cost of hip fracture at $27,527 in 1997 dollars (CPI adjusted to $42,942 in 2010 dollars) [5], much lower than our total direct costs ($52,232 to 54,289, 2010 dollars). Differences in cost estimates likely reflect changes in health-care system costs over time as well as variations in study designs. Primarily, authors relied on patient chart review and interviews to estimate resource utilization among hip fracture patients in a single Ontario region, with our analysis providing a more comprehensive estimate based on actual resource utilization for hip fractures across Ontario. Although total costs are useful, attributable costs provide greater clinical implication for health policy decision-making as it adjusts for costs of typical health-care resource use among similar, non-hip fracture individuals [24, 26]. The 1-year direct cost of hip fracture among women from three regions in Québec was estimated to be $46,664 in 2009 dollars ($47,804 in 2010 dollars). This estimate is closer to our total direct mean cost estimate ($52,232 among women), yet is limited by not including a control group to permit the identification of attributable costs of hip fractures, or considering men [7]. Fracture costs were recently estimated using provincial data from Manitoba [6]. Although this study was comprehensive by estimating the median attributable costs of several types of fracture (hip, wrist, humerus, and a group of other fractures), it was limited in its ability to incorporate costs associated with specific home care, rehabilitation, or emergency department services. Authors estimated the 1-year median direct attributable costs by subtracting pre-fracture costs from post-fracture costs with attributable hip fracture costs estimates of $20,129 in women and $19,330 in men (2006 dollars) after adjustment [24], which are substantially lower than our mean estimates of $36,929 in women and $39,479 in men. Our study reports mean attributable costs, the metric used in cost-effectiveness analyses [27, 28], whereas the Manitoba study provides median costs. Collectively, these methodological variations may explain cost differences between our studies.

Our study is also unique by providing attributable costs associated with residence in LTC and survival, as well as costs and health-care utilization in the second year. Indeed, we found that attributable hip fracture costs were higher for individuals living in the community at the time of fracture—related to the large proportion of community-dwelling seniors that relocate to LTC post-hip fracture. Our results may thus be readily applied to inform cost-effectiveness analyses based on interventions among residents in long-term care and those residing in the community.

Costing analyses are often difficult to generalize between countries due to differences in actual costs, health-care systems, and treatment patterns. However, the substantial costs, low rates of post-fracture screening and treatment, and mortality subsequent to hip fractures reported in our study are comparable to other countries [29–31]. A recent systematic review of US health-care costs identified the US hip fracture hospitalization unit cost to range from $8,358 to 32,195 US dollars and medical costs to range between $15,294 and 71,272 US dollars [29]. Of greatest clinical concern is the loss of independence and mortality risk following hip fracture and low treatment rates. Our findings are consistent with prior estimates [1, 31–34] and emphasize the urgent need to better manage osteoporosis and develop targeted interventions to reduce hip fracture risk. We found that only 10 % (men) to 32 % (women) of patients filled an osteoporosis treatment prior to fracture, and this increased only to 22 % of men and 44 % of women within the year after hip fracture. The Ontario Ministry of Health and Long-Term Care funded a post-fracture care strategy that started to screen patients in fracture clinics in 2007 and an intervention among small community hospitals in 2008—both aim to improve post-fracture osteoporosis management [35, 36]. Post-fracture testing and treatment rates may thus have improved in recent years, and our results may inform cost-effectiveness analyses of interventions to reduce hip fracture risk. We identified that 24 % of women and 19 % of men living in the community at the time of fracture entered a long-term care facility, and 22 % of women and 33 % of men died within the first year following hip fracture. Our results also identify that death remained elevated into the second year post-fracture, a finding previously been shown to persist for up to 5 to 10 years post-fracture [3, 32, 37]. However, the underlying contribution of fracture vs. underlying frailty towards mortality post-hip fracture remains uncertain. While there is a growing body of literature evaluating sex-related differences in osteoporosis [38, 39], understanding sex differences in mortality following hip fractures warrants further study.

There are study limitations worth noting. First, although our hip and non-hip fracture cohorts were well matched, matching could only be achieved based on observed variables. Unmeasured factors such as frailty could be associated with hip fracture risk and subsequent health-care utilization and mortality. We therefore may have overestimated the attributable costs associated with hip fracture by insufficient matching on underlying frailty. Second, while there is a significant value in health-care utilization data to estimate health-care resource use, it is possible that some hip fractures or costs were not identified. Nonetheless, hip fracture hospitalization codes are one of the most reliable hospital diagnoses [9], and overall database validity has been thoroughly described in literature [15]. Prescription drug costs may also be underestimated as drugs dispensed in hospital are not captured in the ODB pharmacy claims; however, they are accounted for in the cost per weighted hospital case and thus included in the hospitalization cost. We thus anticipate that our study is comprehensive and provides a good estimate of the direct health-care costs attributed to hip fractures. However, we also acknowledge that by using a health-care payer perspective, patient costs, such as prescription co-pay and patient-specific costs for LTC accommodation were not considered.

Major study strengths include our comprehensively matched non-hip fracture cohort and analyses reported by age, sex, and residence status. We identified significant health-care costs, entry into LTC, and mortality attributed to hip fractures. As our population ages, the number of hip fractures is estimated to increase [4]. Unless resources are allocated toward the prevention and efficient management of hip fractures, these fractures will increasingly become a major burden to our health-care system. Our results provide a framework to inform future research into the health and economic impact of osteoporotic fractures, and data can be readily used in cost-effectiveness analyses. Our results are particularly timely as new osteoporosis treatments enter the market and we examine interventions to reduce hip fracture risk among seniors.

## Appendix

**Table 5:** Health resource utilization and outcomes in second year after hip fracture compared to matched non-hip fracture cohort, by sex

	Females			Males		
	Percent hip fracture cohort (*N* = 22,418)	Percent non-hip fracture cohort (*N* = 22,418)	Percent attributable	Percent hip fracture cohort (*N* = 7,611)	Percent non-hip fracture cohort (*N* = 7,611)	Percent attributable
Resource utilization						
Acute hospitalizations	19.3	16.9	2.4^*^	20.7	19.5	1.2
Same day surgeries	8.6	11.5	−2.9^*^	11.2	17.2	−6.0^*^
Emergency visits	32.1	36.6	−4.5	30.6	33.8	−3.2^*^
Complex continuing care	1.5	1.0	0.5^*^	2.1	1.1	0.6^*^
Rehabilitation	1.8	1.1	0.7^*^	1.5	0.9	0^*^
Long-term care	32.1	22.2	9.9^*^	22.2	16.9	5.3^*^
Community at index	23.8	7.3	16.5^*^	17.0	5.3	11.7^*^
Home care	29.1	23.6	5.5^*^	24.5	19.5	5.0^*^
Physician services	76.5	85.0	−8.5^*^	65.2	83.7	−18.5^*^
DXA test	6.6	8.8	−2.2^*^	3.3	1.9	1.4^*^
Prescriptions	75.6	84.0	−8.4^*^	63	81.6	−18.6^*^
Osteoporosis treatment	37.0	26.1	10.9^*^	16.6	6.2	10.4^*^
Opioids	27.4	24.7	2.7^*^	22.7	21.7	1.0
NSAIDs	13.8	19.5	−5.7^*^	11.7	18.7	−7.0^*^
Health outcomes						
Second hip fracture	1.7	0	1.7	1.4	0	1.4^*^
Death (overall)	9.1	8.3	0.8^*^	11.3	9.4	1.9^*^
Age group						
66–69	4.8	1.7	3.1^*^	7.8	1.7	6.1^*^
70–74	5.6	2.7	2.9^*^	8.4	3.9	4.5^*^
75–79	7.7	4.9	2.8^*^	10.2	6.7	3.5^*^
80–84	8.2	6.4	1.8^*^	11.7	10.2	1.5
85–89	10.2	9.8	0.4^*^	12.6	12.8	−0.2
90+	12.5	14.9	−2.4^*^	14.4	15.7	−1.3
LTC at index	12.4	17.2	−4.8^*^	14.2	19.7	−5.5^*^
Community at index	8.2	5.8	2.4^*^	10.7	7.1	3.6^*^

*Attributable* percentage of hip fracture patients − percentage of non-hip fracture patients, *LTC* long-term care, *NSAID* nonsteroidal anti-inflammatory drug

***** *p* < 0.05 (significant at this level)

**Table 6:** Mean total and attributable direct health-care costs (2010 Canadian dollars) in second year after index date among in the hip fracture and non-hip fracture cohorts, by sex

Resource type	Females (*N* = 22,418)				Males (*N* = 7,611)			
	Hip fracture	Non-hip fracture	Attributable (95 % CI)	%	Hip fracture	Non-hip fracture	Attributable (95 % CI)	%
Acute hospitalizations	2,988	2,414	574 (388, 771)	12	3,889	3,104	785 (347, 1247)	25
Same day surgeries	107	141	−33 (−44, −23)	0	133	211	−78 (−99, −58)	0
Emergency visits	266	255	11 (0, 21)	0	292	285	7 (−14, 28)	0
Complex continuing care	372	197	174 (104, 244)	4	532	174	358 (229, 485)	23
Rehabilitation	343	246	97 (37, 151)	2	297	177	120 (30, 209)	4
Long-term care	9,569	6,356	3,213 (2,984, 3,435)	70	6,202	4,627	1,575 (1,188, 1,877)	51
Home care	1,284	919	364 (302, 429)	8	1,180	649	531 (427, 641)	17
Physician services	1,320	1,292	27 (−4, 59)	0	1,365	1,484	−120 (−186, −49)	0
Prescription Medications	2,085	1,913	171 (130, 214)	4	1,757	1,853	−95 (−172, −22)	0
Total mean cost/year	18,333	13,734	4,599 (4,233, 4,972)	100	15,648	12,610	3,083 (2,334, 3,764)	100
Age group								
66–69	15,283	6,840	8,442 (6,434, 10,414)		14,470	6,738	7,732 (5,139, 10,298)	
70–74	16,106	8,785	7,321 (6,049, 8,615)		15,920	10,504	5,416 (3,047, 7,779)	
75–79	18,213	11,695	6,518 (5,571, 7,445)		17,866	12,493	5,373 (3,708, 7,206)	
80–84	18,758	14,092	4,666 (3,953, 5,420)		16,379	13,170	3,209 (1,901, 4,559)	
85–89	19,554	15,566	3,988 (3,198, 4,758)		14,852	13,755	1,097 (−303, 2,479)	
90+	17,841	15,944	1,897 (1,093, 2,691)		12,250	14,661	−2,411 (−4,394, −449)	

*Attributable* mean cost hip fracture cohort − mean cost non-hip fracture cohort, *CI* confidence interval
